# Episodic Frequency of Energy-Dense Food Consumption in Women with Excessive Adiposity

**DOI:** 10.1155/2017/5910174

**Published:** 2017-11-08

**Authors:** Antonio Laguna-Camacho, Gustavo A. Castro-Nava, Jerónimo A. López-Arriaga

**Affiliations:** Medical Sciences Research Centre, Autonomous University of the State of Mexico, Toluca, MEX, Mexico

## Abstract

Each episode of fatty or sugary food consumption contributes to the pathophysiological alterations found in obesity. The present study estimated episodic frequency of energy-dense food (EDF) consumption in 348 adult women with excessive adiposity. Participants reported in open questions their habitual exercise and EDF consumption per week. Body fat percentage was measured using electric impedance analysis. Variations in EDF consumption by age, fat mass, and exercise levels were examined by factorial analysis of variance. The frequency of consumption of EDF was on average 12 times per week and it did not vary significantly across subgroups. It is argued that, to reduce obesity and its comorbidities, lowering a high episodic frequency of EDF consumption could be recommended in clinical settings.

## 1. Introduction

The high prevalence of obesity and unhealthy eating habits is a public health concern due to its association with chronic illnesses [1]. For instance, hypertrophy of adipocytes by lipidic overload generates inflammation of the adipose tissue [2, 3]. Each bout of excessive intake also inflames body tissues with a postprandial peak of circulating fatty acids and glucose [4–7]. Thus, excessive adiposity and continuous episodes of energy-dense food (EDF) consumption would contribute to the pathophysiology found in obesity [8].

In psychology the frequency of episodes of any behaviour is used to examine the consequences of engaging repeatedly in such behaviour [9]. Food frequency questionnaires (FFQs) used in epidemiology convert the frequency of food intake into energy and nutrient estimates validated against reported intakes of deficient accuracy [10–12]. There is little research that examines the frequency of eating behaviour without conversion to physical estimations.

Some investigations have paid attention to the frequency of consumption of high-calorie food. For example, higher frequency of eating fast food is associated with poorer nutrition intake and higher body mass index [13–15]. A study classified high consumption of takeaway meals as more than four times per week [16], similar to another study that observed an average consumption of five fast-food meals per week [17]. A large epidemiological study reported also bivariate associations between frequency of intake of various types of food and weight change [18]. Such investigations, however, fail to cover together all the episodes of EDF consumption for calculating a total frequency.

To start addressing this gap, a proposal is to focus on the single habit of eating EDF in daily life. People recognise well foods high in fat and sugar [19]. Therefore, a single category of EDF can include the most common fatty and sugary foods that would also represent other similar food items not covered. In this way, the number of times that people carry out that specified behavior pattern over a time period could be estimated.

The present work examined such episodic frequency of habitual EDF consumption in Mexican adult women with excess of adiposity. The variations in the frequency of episodes of EDF consumption were additionally examined in terms of individual characteristics.

## 2. Method

### 2.1. Participants

Participants were adult women who received recommendations for a healthy weight at the Medical Sciences Research Centre (MCRC) of the Autonomous University of the State of Mexico, in Toluca city, Mexico. Participants were recruited among the general public through a notice posted at the gate of the MCRC. The present study examined data of 348 women who met the eligibility criteria: age between 18 and 57 years old, body fat ≥ 30% [20], and no chronic ill health. Volunteers provided signed consent and the institutional ethics board approved the protocol.

### 2.2. Procedure and Measures

Participants reported orally in open questions their motives to lose weight, demographic information, and the times per week that they habitually consumed EDF and exercised.

The question repeated to obtain the frequency of EDF consumption at breakfasts, between breakfasts and lunch, at lunch, between lunch and breakfast, at dinner, and after dinner was “Taking as reference the past four weeks, how many times a week on average do you consume foods high in calories [time of the day]? Examples of high calorie foods are [list of examples].” The question to obtain the frequency of exercise was “Taking as reference the preceding four weeks, how many times a week on average do you take exercise? Examples of exercise are [list of examples].” Popular examples of foods high in fat and/or sugar and of exercise collected previously in the same location were given to participants after asking them for the frequency of each habit (Table 1). To elicit a specific number of EDF or exercise episodes per week, no answer options with frequency ranges were given. The total frequency of EDF consumption a week was calculated summing up the individual frequencies of EDF consumption at all the times of the day.

Height, weight, and body fat percentage were taken using a wall stadiometer (Seca 1013522) and a body composition analyser with bioelectrical impedance (Tanita BF541) with the participant wearing light clothing and barefoot. Body mass index was calculated dividing weight in kilograms by squared height in meters.

### 2.3. Analysis of Data

The responses of occupation and motives to lose weight were put into categories on the basis of conceptual similarity. The proportion of responses in each category was calculated dividing the number of times that it was reported by the (sub)total number of participants. To study the frequency of EDF consumption according to the characteristics of participants, subgroups were formed by levels of age, fat mass, and exercise. Since the endocrine regulation that potentially influences energy exchange as well as body composition changes over adult life [21], age was split at the age of 38 y when such changes accelerate (younger participants < 38 y and older participants ≥ 38 y). Because the level of adiposity could also to some extent impact intake and expenditure [3], levels were considered at about the median of body fat percentage (lower adiposity < 40% of FM and higher adiposity ≥ 40% FM), which agrees with cut-off points reported elsewhere [20]. Energy input and output could be additionally influenced by exercise [22, 23]; thus, the frequency of exercise was classified considering scientific recommendations (low exercise < 3 times per week and high exercise ≥ 3 times per week; [24]). The association of age, fat mass, and exercise levels with frequency of EDF consumption was examined by factorial analysis of variance.

## 3. Results

Overall, half of participants were paid employees, more than a quarter of participants were students, and fewer than a quarter reported home as occupation (Table 2). Proportions of employed participants were similar between younger and older participants, but more younger participants were students, and more older participants were housewives (Table 2). There was no difference in occupation between participant subgroups in terms of fat mass or exercise levels. The most frequently reported reasons to lose weight were health, physical problems, and appearance, with no differences by age, fat mass, or exercise levels.

Participants had on average a frequency of 1.5 episodes of exercise per week (Table 2). They had also an average frequency of EDF consumption of 12 episodes of per week (Table 2). There were no reliable differences in frequency of EDF consumption by levels of age, fat mass, and exercise. Younger women had marginally higher frequency of EDF consumption than older women (age main effect: _*p*_*η*^2^ = 0.009, *F* = 3.26, and *p* = 0.07). There was no evidence that frequency of EDF consumption varied by levels of adiposity or exercise (fat mass main effect: _*p*_*η*^2^ = 0.002, *F* = 0.67, and *p* = 0.41; exercise main effect: _*p*_*η*^2^ = 0.005, *F* = 1.67, and *p* = 0.2).

**Figure 1:**
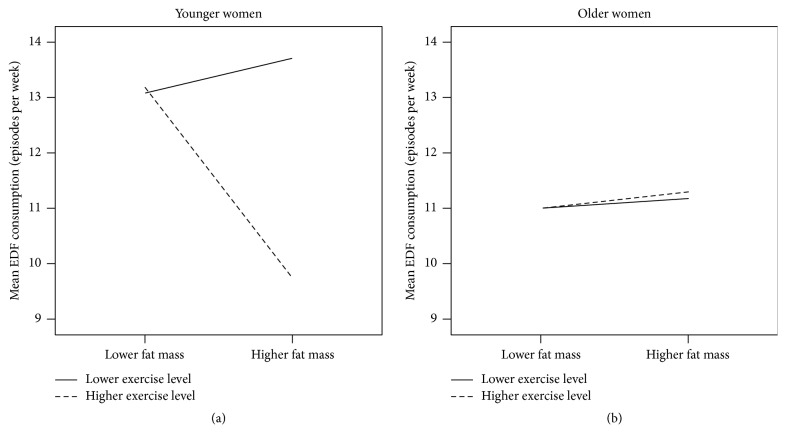
Habitual frequency of energy-dense food (EDF) consumption in participant women with excess of adiposity.

The variations in frequency of EDF consumption by fat mass and exercise levels were also independently examined in younger and older women. In younger women, those with the highest adiposity and the highest levels of exercise tended to show lower frequency of EDF consumption (fat mass effect: _*p*_*η*^2^ = 0.011, *F* = 2.65, and *p* = 0.11; exercise main effect: _*p*_*η*^2^ = 0.021, *F* = 4.91, and *p* = 0.03; fat mass and exercise interaction effect: _*p*_*η*^2^ = 0.023,* F* = 5.48, and *p* = 0.02; Figure 1(a)). In older women, no evidence of differences in frequency of consumption of EDF by fat mass and exercise levels was observed (fat mass effect: _*p*_*η*^2^ = 0.001, *F* = 0.04, and *p* = 0.84; exercise main effect: _*p*_*η*^2^ = 0.001, *F* = 0.01, and *p* = 0.96; fat mass and exercise interaction effect: _*p*_*η*^2^ = 0.001, *F* = 0.01, and *p* = 0.96; Figure 1(b)).

## 4. Discussion

The present study found that the habitual number of times that Mexican adult women with excessive adiposity consume EDF in their daily life was on average 12 times a week. Such level of frequency of EDF consumption is informative about such a relevant pattern of behaviour, the same as the observed average of 1.5 episodes of exercise a week. That is, adult women with excessive adiposity showed overall low frequency of exercise and high frequency of consumption of EDF. Although such data would be common knowledge about major contributors to overweight and obesity [25], the estimates of episodic frequency in which they are expressed provide an innovative behavioural perspective that could be applied to manage habitual EDF consumption.

There are recommendations that individuals should exercise three or more times per week [24]. There is, however, little information about a limit of frequency to prevent adverse effects of EDF consumption on health. The frequency of habits of eating and exercise has been associated with changes in body weight [26]. In addition, small deficits in energy intake could be beneficial for weight loss and metabolic improvement [27, 28]. Hence a recommendation can be formulated such as to decrease by half the usual number of episodes of EDF consumption (i.e., fewer than seven times per week).

The present study found little variability in levels of frequency of EDF consumption across participants. Younger women with higher levels of body fat and of exercise tended to have lower frequency of EDF consumption, which may reflect weight loss efforts by means of restriction of high-calorie food and increased exercise. In line with this possibility, higher prevalence of dieting is reported by young women with higher levels of adiposity [29]. Such kind of differences in baseline behaviour frequency should be considered in habit change interventions in order to prevent sources of bias in outcomes.

There are further possibilities of investigation on the frequency of habits of eating and exercise. A focus of survey research has been on how participants process frequency, that is, how they came up with a number (e.g., [30]), rather than what the frequency of a habit is. A criticism to food frequency questions is that they could depend more on participants' general knowledge of their lifestyle habits than on the actual episodes [10]. This is because most epidemiology studies estimate food frequency over several months (i.e., [18]), which opens possibilities of error by variations in habit frequency over such long interval. In contrast, memory research supports the reliability of reports of recently occurred autobiographical events such as eating occasions [31–33]. The present study estimated the frequency over the preceding four weeks, which would allow participants to exploit their memories of recent occurrences of a habit. Alternatively, episodes of a habit could be recorded as they occur to calculate actual changes in their frequency over time.

An additional issue is to measure with accuracy the amount of intake or dose of expenditure for each episode of eating or exercise, which at present is still no possible [12, 34]. In the current study, the habit of EDF consumption gathered together foods that would contribute to intake from fat and/or sugar at an eating episode, and the habit of exercise gathered common examples that would contribute to a bout of extra expenditure. Nevertheless, future investigations are warranted on methods to improve the estimates of energy ingested or spent at eating or exercise episodes.

## 5. Conclusion

This study reports that Mexican women with excess of adiposity had a usual consumption of food high in sugar and/or fat of 12 times per week. Each eating episode including EDF is likely to cause a physiological adverse peak of glucose and/or fatty acids in the circulation. Thus, to reduce the development of obesity and chronic disease, the current episodic frequency of EDF consumption in women with excess of body fatness could be managed by recommending a modest decrease from high baseline levels.

## Figures and Tables

**Table 1:** Descriptions of studied behavioural patterns^a^.

Habit	Examples given to describe the behaviour
High-calorie food/drink consumption: at breakfast, between breakfast and lunch, at lunch, between lunch and dinner, at dinner, after dinner	*Burger, pizza, hotdog, fries, chips, fatty meat or typical preparations high in fat, desserts, chocolate, ice-cream, sweets, biscuits, pastry, cake, nachos with cheese, crisps, snacks, empanadas, soft drinks, sugary beverages, bottled juice.*
Exercise	*Go to the gym, jog, fitness or dance class, exercise routine at home, go for a walk.*

^a^Translations from Spanish to English language.

**Table 2:** Characteristics of participant women with excess of adiposity^a^.

	All participants	Lower fat mass				Higher fat mass			
		Younger		Older		Younger		Older	
	*N* = 348	Lower exercise *n* = 99	Higher exercise *n* = 39	Lower exercise *n* = 29	Higher exercise *n* = 21	Lower exercise *n* = 76	Higher exercise *n* = 22	Lower exercise *n* = 45	Higher exercise *n* = 17
Age in years	32.9 (19,52)	25.9 (18.0,36.0)	26.6 (18.0,37.0)	46.3 ${\left(38.0,57.0\right)}^{}$	44.3 ${\left(38.0,55.7\right)}^{}$	28.6 ${\left(20.9,37.0\right)}^{}$	27.2 ${\left(20.2,36.7\right)}^{}$	44.9 ${\left(38.0,55.0\right)}^{}$	45.4 ${\left(38.0,56.8\right)}^{}$
Occupation									
*Employee*	49.4%	34.3%	51.3%	65.5%	47.6%	48.7%	45.5%	68.9%	64.7%
*Student*	28.7%	50.5%	41.0%	0.0%	4.8%	31.6%	40.9%	0.0%	0.0%
*Home*	21.8%	15.2%	7.7%	34.5%	47.6%	19.7%	13.6%	31.1%	35.3%
Motives to lose weight									
*Health*	64.9%	42.4%	56.4%	41.4%	66.7%	75.0%	59.1%	75.6%	70.6%
*Physical problems*	28.4%	23.2%	28.2%	48.3%	28.6%	22.4%	27.3%	37.8%	29.4%
*Physical appearance*	20.7%	31.3%	23.1%	13.8%	14.3%	17.1%	13.6%	15.6%	11.8%
*Recent weight gain*	10.9%	10.1%	12.8%	13.8%	19.0%	10.5%	13.6%	4.4%	11.8%
*Family medical antecedents*	2.6%	3.0%	2.6%	0.0%	4.8%	3.9%	4.5%	0.0%	0.0%
BMI kg/m^2^	30.6 (24.4, 39.7)	27.3 (23.7, 31.0)	27.5 (23.9, 31.8)	28.9 (24.9, 33.9)	27.3 (24.4, 31.9)	34.6 (29.3, 43.7)	32.4 (26.9, 41.3)	34.5 (29.5, 41.5)	32.8 (26.3, 40.0)
Body fat percentage	39.4 (31.0, 48.5)	35.2 (30.3, 39.7)	34.6 (31.0, 39.9)	36.9 (30.7, 39.8)	35.2 (29.9, 39.8)	44.5 (40.1, 50.8)	42.9 (40.0, 48.5)	44.7 (40.9, 49.3)	43.6 (40.2, 50.5)
Episodes per week									
*Exercise*	1.5 (0.0, 5.6)	0.4 (0.0, 2.0)	4.4 (3.0, 7.0)	0.3 (0.0, 2.0)	3.9 (3.0, 6.8)	0.5 (0.0, 2.0)	4.3 (3.0, 7.0)	0.0 (0.0, 2.0)	4.8 (3.0, 7.0)
*Energy-dense food/drink*	12.4 (4.0, 24.0)	13.1 (4.0, 24.0)	13.2 (4.0, 25.0)	11.0 (2.5, 19.5)	11.0 (2.1, 25.5)	13.7 (3.9, 26.0)	9.7 (0.8, 17.9)	11.2 (2.3, 23.4)	11.3 (3.0, 23.5)

^a^Age, BMI, body fat percentage, and episodes per week of specified behaviour are expressed as means and 95% confidence interval in brackets. Occupation and motives to lose weight are expressed as proportion of participants.
